# Knowledge, Attitude and Practice Toward Needle Stick Injury Among Health Care Workers in Mogadishu Somalia

**DOI:** 10.1002/hsr2.72966

**Published:** 2026-08-04

**Authors:** Abdullahi Hassan Elmi, Mohamed Mustaf Ahmed, Mulki Mukhtar Hassan, Najib Isse Dirie

**Affiliations:** ^1^ Department of Nursing and Midwifery, Dr. Sumait Hospital SIMAD University Mogadishu Somalia; ^2^ Faculty of Medicine and Health Sciences SIMAD University Mogadishu Somalia; ^3^ Dr. Sumait Hospitals, Faculty of Medicine and Health Sciences SIMAD University Mogadishu Somalia; ^4^ Department of Urology, Dr. Sumait Hospitals, Faculty of Medicine and Health Sciences SIMAD University Mogadishu Somalia

**Keywords:** attitude, healthcare workers, knowledge, needle stick injury, occupational safety, practice, Somalia

## Abstract

**Background:**

Needlestick injuries (NSIs) remain a major occupational hazard among healthcare workers (HCWs), increasing the risk of exposure to blood‐borne infections such as hepatitis B virus (HBV), hepatitis C virus (HCV), and human immunodeficiency virus (HIV). Although largely preventable, NSIs continue to occur frequently in fragile and resource‐limited health systems, including Somalia. This study assessed healthcare workers’ knowledge, attitudes, and practices regarding NSIs in Mogadishu, Somalia, to identify existing gaps and inform occupational safety interventions.

**Methods:**

A facility‐based cross‐sectional study was conducted in Mogadishu, Somalia, from July 2024 to January 2025. A total of 422 healthcare workers from public and private health facilities were selected using stratified random sampling. Data were collected through a validated self‐administered electronic questionnaire covering sociodemographic characteristics and knowledge, attitudes, and practices related to NSIs. Scores for knowledge, attitude, and practice were categorized using Bloom's cut‐off criteria. Data were analyzed using descriptive statistics and multivariable logistic regression in SPSS version 20, with statistical significance set at *p* ≤ 0.05.

**Results:**

Overall, 82.94% of participants demonstrated good knowledge of needlestick injuries (NSIs), with a mean score of 8.15 ± 2.23. However, gaps remained in awareness of post‐exposure prophylaxis (PEP) and the recommended timing of follow‐up viral testing. Attitudes toward NSI prevention were generally positive, with 96.21% of participants classified as having a positive attitude. Nevertheless, some respondents continued to prioritize patient care over healthcare worker safety. Preventive practices were less satisfactory, with only 44.79% of participants reporting good practice. Underreporting of injuries and delayed uptake of PEP were also notable concerns. In the adjusted analysis, healthcare workers aged 18–30 years were less likely to report good preventive practices than those aged over 40 years (AOR = 0.30, *p* = 0.001). In contrast, nurses (AOR = 3.29, *p* = 0.002) and specialists (AOR = 2.67, *p* = 0.048) were more likely to report good preventive practices than laboratory technicians. Estimates from the attitude model were unstable because of sparse outcome data and should therefore be interpreted cautiously.

**Conclusion:**

Despite relatively high knowledge and generally positive attitudes toward NSI prevention, important gaps in preventive practice remain among healthcare workers in Mogadishu. Underreporting of injuries and inadequate post‐exposure management highlight a persistent gap between awareness and action. Targeted interventions aimed at younger healthcare workers, improved reporting systems, better access to PEP, and stronger institutional safety practices are needed to reduce the burden of NSIs in resource‐limited settings.

## 1 Introduction

Globally, the healthcare workforce comprises approximately 35 million individuals, accounting for nearly 12% of the total working population [1]. Each year, an estimated 2 million healthcare workers experience occupational injuries, and nearly 3 million sustain percutaneous exposure to blood‐borne viruses (BBVs) [1]. These exposures are associated with approximately 16,000 cases of hepatitis C virus (HCV), 66,000 cases of hepatitis B virus (HBV), and between 200 and 5000 cases of human immunodeficiency virus (HIV) infections among healthcare personnel annually [2]. Such figures underscore the substantial occupational risks faced by healthcare workers worldwide.

Needlestick injuries (NSIs) represent one of the most common and serious occupational hazards in healthcare settings, despite being largely preventable. Even a single injury may transmit more than 20 pathogenic organisms, most notably HBV, HCV, and HIV [2, 3]. NSIs are typically defined as puncture wounds caused by sharp medical instruments, including hypodermic needles, intravenous cannulas, and fluid collection devices. These injuries often occur during routine clinical activities such as specimen collection, needle recapping, improper disposal of sharps, or unsafe handling practices [3].

The risk of NSIs and subsequent BBV transmission can be substantially reduced through evidence‐based preventive strategies, including HBV vaccination, adherence to standard precautions, appropriate disposal of sharps, and timely post‐exposure prophylaxis (PEP) [4]. Nevertheless, unsafe injection practices remain a significant global public health problem. It is estimated that contaminated injections contribute annually to 8–16 million HBV infections, 2.4–4.5 million HCV infections, and approximately 80,000–160,000 HIV infections worldwide [5]. The World Health Organization (WHO) further estimates that between 2000 and 2030, sharps‐related HCV infections alone will result in approximately 142 premature deaths, while HBV infections attributable to sharps injuries may cause more than 260 early deaths [5, 6]. In addition, unsafe injections in developing and transitional countries have been reported to account for 5% of new HIV infections, 32% of new HBV infections, and 40% of new HCV infections [7, 8].

Healthcare workers (HCWs) include all individuals engaged in activities aimed at improving health outcomes, encompassing direct care providers such as physicians, nurses, laboratory technicians, and pharmacists, as well as health management and support personnel, including administrative staff, cleaners, drivers, and other ancillary workers [9]. In low‐income and fragile health systems, HCWs often face heightened occupational risks due to limited access to safety‐engineered devices, inadequate infection‐prevention infrastructure, and weak occupational health services.

In this study, the Knowledge, Attitude, Practice (KAP) framework was adopted as a conceptual model to examine healthcare workers’ responses to NSIs. Knowledge refers to healthcare workers’ factual understanding of NSI risks, routes of BBV transmission, and appropriate post‐exposure actions. Attitude reflects their perceptions, beliefs, and feelings toward NSI prevention, reporting, and occupational safety. Practice denotes the routine behaviors and preventive actions undertaken to minimize exposure, such as safe handling and disposal of sharps and timely reporting of injuries. Within this framework, post‐exposure prophylaxis (PEP) is defined as the prompt administration of preventive medical treatment following potential occupational exposure to BBVs to reduce the risk of infection [9].

Studies from several low‐ and middle‐income countries have shown that healthcare workers often have general awareness of the risks associated with needlestick injuries, but important gaps remain in reporting practices, post‐exposure management, and consistent adherence to preventive measures [1, 2, 3, 4]. These studies have provided useful evidence on knowledge, attitudes, and practices related to needlestick injury prevention, but their findings may not be fully transferable to Somalia, where healthcare delivery takes place within a fragile health system characterized by limited occupational health services, constrained infection‐prevention resources, and weak institutional reporting structures.

In Somalia, evidence regarding needlestick injuries among healthcare workers remains limited. The small number of available studies from Mogadishu has mainly focused on the occurrence, prevalence, and exposure patterns of needlestick and sharps injuries. However, little is known about healthcare workers’ knowledge of prevention and post‐exposure management, their attitudes toward occupational safety, their reporting behavior after injury, and their routine preventive practices in the workplace. Addressing these gaps is important for understanding not only the frequency of occupational exposure, but also the behavioral and institutional factors that may influence prevention and response. This study, therefore, aimed to assess the knowledge, attitudes, and practices of healthcare workers regarding needlestick injuries in Mogadishu, Somalia, and to generate context‐specific evidence that can inform targeted occupational safety strategies in a resource‐limited setting.

## 2 Methods

### 2.1 Study Design and Study Area

A cross‐sectional study was conducted in Mogadishu, Somalia, between July 2024 and January 2025. Mogadishu, the capital and most densely populated city in the country, hosts a wide range of public and private healthcare facilities that serve diverse populations from different socioeconomic backgrounds. The study was conducted in both public and private healthcare facilities, including hospitals, clinics, and specialized centers, to ensure representation of different levels of healthcare service delivery. Mogadishu was purposively selected as the study site because it represents the largest concentration of healthcare institutions in Somalia and reflects the operational challenges of healthcare delivery in a fragile, resource‐limited health system. This approach allowed for the inclusion of healthcare workers from diverse professional backgrounds and institutional settings, thereby enhancing the representativeness and contextual relevance of the study.

### 2.2 Study Population and Eligibility Criteria

The study population consisted of healthcare providers working in public and private healthcare facilities in Mogadishu, Somalia. Inclusion criteria were: (i) healthcare providers aged 18 years or older; (ii) employment in a healthcare facility located in Mogadishu at the time of data collection; (iii) direct or indirect involvement in patient care, including nurses, physicians, midwives, laboratory technicians, and other allied healthcare professionals; and (iv) willingness to participate in the study and provide informed consent. Exclusion criteria included healthcare providers who were not Somali citizens, those who were not actively working during the data collection period, individuals unwilling to participate, and those who declined or failed to provide informed consent.

### 2.3 Sample Size and Sample Technique

The sample size was calculated using a single population proportion formula, assuming a prevalence of 50% for poor knowledge regarding needlestick injuries, with a 95% confidence interval and a 5% margin of error. The assumption of a 50% prevalence was used as a conservative estimate to maximize the sample size in the absence of reliable, context‐specific prevalence data from Somalia. This approach is widely recommended in epidemiological research when prior prevalence estimates are unavailable or highly variable, as it ensures adequate statistical power and precision [10].

### 2.4 Research Instruments and Measurements

Data were collected using a structured, self‐administered questionnaire developed based on relevant literature and international guidelines and reviewed by three external subject‐matter experts. The questionnaire consisted of four main sections: (i) sociodemographic characteristics, (ii) knowledge related to needlestick injuries, (iii) attitudes toward needlestick injury prevention, and (iv) preventive practices.

The knowledge section included statements with response options of true, false, or not sure. Correct responses were assigned a score of 1, while incorrect or “not sure” responses were scored as 0. Knowledge scores were converted into percentages and categorized as poor (≤ 60%) or good (> 60%) according to Bloom's cut‐off point classification [11, 12, 13].

The attitude section assessed participants’ perceptions and beliefs regarding needlestick injury prevention using a 5‐point Likert scale ranging from strongly disagree (1 point) to strongly agree (5 points). Negatively worded items were reverse scored to ensure consistency. Overall attitude scores were calculated and categorized as negative (≤ 60%) or positive (> 60%) based on Bloom's criteria [11, 12, 13, 14, 15, 16].

The practice section evaluated participants’ self‐reported preventive behaviors using frequency‐based response options (always, occasionally, and never). Responses indicating correct practice (always) were assigned a score of 1, while occasionally and never were scored as 0. Practice scores were similarly converted into percentages and classified as poor (≤ 60%) or good (> 60%) using Bloom's cut‐off point classification [11, 12, 13, 14, 15, 16].

The questionnaire was initially developed in English and subsequently translated into Somali to ensure clarity and cultural appropriateness. Content validity was assessed using the Item Objective Congruence method with feedback from three external experts [17]. A pilot study involving 30 healthcare workers was conducted prior to the main data collection to assess the clarity, relevance, and comprehensiveness of the questionnaire. The pilot testing evaluated the wording of items, response options, logical flow of the questionnaire, and the time required for completion. Based on feedback from pilot participants, minor modifications were made to improve item clarity and reduce ambiguity, including rephrasing selected questions and refining response categories. The internal consistency of the questionnaire was assessed using Cronbach's alpha coefficient, which yielded a value of 0.79, indicating good reliability of the instrument for assessing knowledge, attitudes, and practices related to needlestick injuries.

### 2.5 Data Collection Techniques

Data were collected from healthcare workers employed in selected public and private healthcare facilities in Mogadishu, Somalia. Data collection was conducted within the participating facilities during routine working hours.

A structured, validated electronic questionnaire was used for data collection. The questionnaire was distributed to eligible participants using electronic devices, allowing respondents to complete the survey independently at their convenience. This approach was adopted to enhance privacy, minimize interviewer bias, and encourage honest responses.

Healthcare facility administrators and unit supervisors assisted in facilitating access to participants by informing eligible healthcare workers about the study and inviting them to participate. Prior to completing the questionnaire, participants were provided with information about the study objectives, assured of confidentiality, and informed that participation was voluntary. Only healthcare workers who provided informed consent were included in the study.

## 3 Data Analysis

The collected data were carefully cleaned, coded, and entered into a spreadsheet before being imported into SPSS version 20 (SPSS Inc., Chicago, IL, USA) for analysis. Data cleaning procedures included checking for completeness, identifying duplicate entries, and verifying the consistency and accuracy of responses prior to statistical analysis. Descriptive statistics were used to summarize the characteristics of the study population, with categorical variables presented as frequencies and percentages and continuous variables reported as means and standard deviations (SD).

For the knowledge domain, correct responses were assigned a score of 1, while incorrect or “not sure” responses were scored as 0. Knowledge scores were converted into percentages and classified as poor (≤ 60%) or good (> 60%) according to Bloom's cut‐off point classification [11, 12, 13]. The attitude domain was assessed using a five‐point Likert scale ranging from strongly disagree (1 point) to strongly agree (5 points), with reverse scoring applied to negatively worded items. Attitude scores were similarly categorized as negative (≤ 60%) or positive (> 60%) based on Bloom's criteria [11, 12, 13, 14, 15, 16]. For the practice domain, responses indicating appropriate behavior (always) were assigned a score of 1, while responses of occasionally or never were scored as 0. Practice scores were converted into percentages and classified as poor (≤ 60%) or good (> 60%) following Bloom's classification [11, 12, 13, 14, 15, 16].

Univariate logistic regression analyses were initially performed to examine associations between independent variables and the study outcomes (knowledge, attitudes, and practices). Variables with a *p*‐value ≤ 0.25 in the univariate analysis and variables considered epidemiologically relevant based on prior literature were considered for inclusion in the multivariable logistic regression models to control for potential confounding. Variables with sparse data or zero‐cell counts were carefully reviewed before inclusion in the multivariable logistic regression models. Where categories contained very small numbers, conceptually similar groups were merged to improve model stability and avoid unreliable estimates. Model goodness of fit was assessed using the Hosmer–Lemeshow test. In the final multivariable analysis, adjusted odds ratios (AORs) with 95% confidence intervals (CIs) were reported, and variables with a *p*‐value ≤ 0.05 were considered statistically significant.

## 4 Results

### 4.1 Sociodemographic Characteristics of the Study Participants

The sociodemographic characteristics of the participants are presented in (Table 1). Overall, the study population was predominantly composed of healthcare workers aged 31–40 years, females, and individuals holding a bachelor's degree. Nurses formed the largest professional group, and most participants had fewer than 5 years of work experience. The majority also reported a monthly income of 200–500 USD.

**Table 1:** Sociodemographic characteristics of the study participants.

Variable	Frequency (*N*)	Percent (%)
Age		
18–30	94	22.27
31–40	273	64.69
> 40	55	13.03
Mean ± SD	31.09 ± 8.79	
Sex		
Male	176	41.71
Female	246	58.29
Level of education		
Diploma	20	4.74
Bachelor's degree	289	68.48
Master's degree	100	23.70
PhD	13	3.08
Professional field		
Specialist	79	18.72
General practitioner	77	18.25
Nursing	181	42.89
Midwifery	36	8.53
Laboratory technician	49	11.61
Years of experience		
0–5	283	67.06
6–10	115	27.25
> 10	24	5.69
Mean ± SD	5.05 ± 3.29	
Monthly income (USD)		
200–500	292	69.19
501–1000	57	13.51
> 1000	73	17.30
Mean ± SD	797.57 ± 930.11	

*Note:* Table 1 presents descriptive characteristics of the study participants. Statistical significance testing was not performed for these variables.

#### 4.1.1 Knowledge of Needle Stick Injury

Overall, participants demonstrated a high level of knowledge regarding needlestick injuries (Table 2). Knowledge was particularly strong in relation to hepatitis B vaccination and the risk of blood‐borne virus transmission. However, important gaps remained in awareness of post‐exposure prophylaxis and the appropriate timing of follow‐up testing after exposure.

**Table 2:** Knowledge regarding needlestick injuries among healthcare workers in Mogadishu, Somalia.

Level of knowledge	Frequency, *N* (%)	Mean ± SD
Good Knowledge (> 60%)	350	(82.94)
	8.15 ± 2.23	
Poor Knowledge (≤ 60%)	72	(17.06)

**Knowledge statements**	**Correct answer *n* (%)**	**Wrong answer *n* (%)**
It's important to attend a biosafety course.	366	(86.73)
	56	(13.27)
It's important to have Hepatitis B virus vaccination.	386	(91.47)
	36	(8.53)
It's important to know my anti‐HBs antibodies level	372	(88.15)
	50	(11.85)
Post‐exposure prophylaxis is required after a needlestick injury.	307	(72.75)
	115	(27.25)
It is important to have occupational health services in the hospital.	356	(84.36)
	66	(15.64)
Hepatitis B virus infection can be transmitted through a needlestick injury.	369	(87.44)
	53	(12.56)
Hepatitis C virus infection can be transmitted through a needlestick injury.	350	(82.94)
	72	(17.06)
HIV infection can be transmitted through a needlestick injury.	358	(84.83)
	64	(15.17)
After a needlestick injury involving an HCV‐infected patient, HCV RNA PCR testing should be performed at 6 weeks.	278	(65.88)
	144	(34.12)
After a needlestick injury involving an HCV‐infected patient, HCV antibody testing should be performed at 4–6 months.	299	(70.85)
	123	(29.15)

#### 4.1.2 Attitudes Toward Needle Stick Injury

Attitudes toward needlestick injury prevention were generally positive among the participants (Table 3). Most respondents expressed concern about sustaining a needlestick injury and recognized that such injuries are preventable and should be reported promptly. Nevertheless, a considerable proportion still perceived patient care as more important than healthcare worker safety, suggesting a potential attitudinal barrier to safer practice.

**Table 3:** Attitudes toward needlestick injury prevention among healthcare workers in Mogadishu, Somalia.

Level of attitude	Frequency, *N* (%)			Mean ± SD	
Positive Attitude (> 60%)	406 (96.21)			21.24 ± 3.04	
Negative Attitude (≤ 60%)	16 (3.79)				

**Attitude statements**	**Strongly agree, *n* (%)**	**Agree, *n* (%)**	**Neutral, *n* (%)**	**Disagree, *n* (%)**	**Strongly disagree, *n* (%)**
I worry about having needle stick injury	266 (63.03)	107 (25.36)	26 (6.16)	16 (3.79)	7 (1.66)
Because sharps disposal containers are not changed often enough where I work, I am concerned about getting a sharps injury	190 (45.02)	142 (33.65)	38 (9.00)	47 (11.14)	5 (1.18)
Patient care is more important than the safety of health care workers.	192 (45.50)	114 (27.01)	38 (9.00)	56 (13.27)	22 (5.21)
All sharps injuries at work should be reported immediately.	227 (53.79)	148 (35.07)	32 (7.58)	15 (3.55)	0.00 (0)
Needlestick injuries are preventable.	223 (52.84)	152 (36.02)	25 (5.92)	19 (4.50)	3 (0.71)

#### 4.1.3 Practice Regarding Needle Stick Injury

Preventive practices related to needlestick injuries were less satisfactory than knowledge and attitudes (Table 4). Although glove use and seeking care after injury were commonly reported, underreporting of injuries and delayed uptake of post‐exposure prophylaxis remained notable concerns. These findings suggest a gap between awareness of needlestick injury prevention and consistent implementation of safe practices.

**Table 4:** Preventive practices regarding needlestick injuries among healthcare workers in Mogadishu, Somalia.

Level of practice	Frequency, *N* (%)	Mean ± SD
Good practice (> 60%)	189 (44.79)	5.06 ± 2.12
Poor practice (≤ 60%)	233 (55.21)	

**Practical statement**	**Always**	**Occasionally/never**
I use gloves when dealing with needles.	351 (83.18%)	71 (16.82%)
If I have a needle stick injury, my immediate action will be to seek healthcare.	307 (72.75%)	115 (27.25%)
I report the injury if it happens.	285 (67.54%)	137 (32.46%)
It is not important to report injuries if reporting does not change the outcome.	194 (45.97%)	228 (54.03%)
I believed the exposure was unlikely to be infectious.	203 (48.10%)	219 (51.90%)
I considered the exposure insignificant.	223 (52.84%)	199 (47.16%)
I wear gloves at the time of work.	321 (76.07%)	101 (23.93%)
I received post‐exposure prophylaxis promptly after an NSI.	252 (59.72%)	170 (40.28%)

### 4.2 Factors Associated With Knowledge, Attitude, and Practice

Factors associated with knowledge, attitudes, and preventive practices were examined using univariate and multivariable logistic regression analyses, as presented in (Tables 5, 6, 7). In the adjusted analyses, professional category was associated with knowledge, while age and professional category were associated with preventive practices. No clear statistically significant association with attitude was identified after adjustment. However, the attitude model was affected by sparse and zero cell counts because very few participants reported negative attitudes. The estimates from this model should therefore be interpreted cautiously.

**Table 5:** Factors associated with knowledge of needlestick injuries among healthcare workers in Mogadishu, Somalia.

	Knowledge level					
Variables	**Good, *n* (%)**	**Poor, *n* (%)**	OR (95% CI)	*p* value	AOR (95% CI)	*p* value
Age (years)*						
18–30	233 (85.35)	40 (14.65)	0.99 (0.44–2.25)	0.984	0.83 (0.28–2.51)	0.746
31–40	70 (74.47)	24 (25.53)	0.5 (0.21–1.20)	0.119	0.43 (0.15–1.20)	0.106
> 40	47 (85.45)	8 (14.55)	1.00		1.00	
Sex*						
Male	152 (86.36)	24 (13.64)	1.54 (0.90–2.62)	0.115	1.43 (0.75–2.73)	0.283
Female	198 (80.49)	48 (19.51)	1.00		1.00	
Level of education*						
Diploma	8 (40.00)	12 (60.00)	0.42 (0.1–1.74)	0.231	0.49 (0.09–2.72)	0.413
Bachelor's degree	251 (86.85)	38 (13.15)	4.13 (1.28–13.28)	0.017*	3.23 (0.69–15.07)	0.136
Master's degree	83 (83)	17 (17)	3.05 (0.89–10.47)	0.076	2.89 (0.74–11.33)	0.128
PhD	8 (61.54)	5 (38.46)	1.00		1.00	
Professional field*						
Specialist	63 (79.75)	16 (20.25)	0.17 (0.04–0.76)	0.021*	0.19 (0.04–1.08)	0.061
General practitioner	57 (74.03)	20 (25.97)	0.12 (0.03–0.55)	0.006*	0.14 (0.03–0.65)	0.012
Nursing	155 (85.64)	26 (14.36)	0.25 (0.06–1.11)	0.068	0.26 (0.06–1.14)	0.074
Midwifery	28 (77.78)	8 (22.22)	0.15 (0.03–0.75)	0.021*	0.14 (0.03–0.79)	0.026
Laboratory technician	47 (95.92)	2 (4.08)	1.00		1.00	
Years of experience*						
0–5 years	233 (82.33)	50 (17.67)	0.20 (0.03–1.54)	0.122	0.2 (0.02–1.73)	0.144
6–10 years	94 (81.74)	21 (18.26)	0.19 (0.02–1.52)	0.119	0.22 (0.03–1.89)	0.170
> 10 years	23 (95.83)	1 (4.17)	1.00		1.00	
Monthly income (USD)*						
200–500	242 (82.88)	50 (17.12)	1.36 (0.72–2.56)	0.342	0.94 (0.34–2.62)	0.901
501–1000	51 (89.47)	6 (10.53)	2.39 (0.87–6.56)	0.092	1.81 (0.52–6.31)	0.352
> 1000	57 (78.08)	16 (21.92)	1.00		1.00	

*Note:* * Indicates variable.

**Table 6:** Factors associated with attitudes toward needlestick injury prevention among healthcare workers in Mogadishu, Somalia.

Variables	Attitude level		OR (95% CI)	*p* value	AOR (95% CI)	*p* value
	Positive, *n* (%)	Negative, *n* (%)				
Age (years)*						
18–30	257 (94.14)	16 (5.86)	2.39 (0.87–6.56)	0.092	2.89 (0.74–11.33)	0.128
31–40	94 (100.00)	0 (0.00)	1.36 (0.72–2.56)	0.342	1.43 (0.75–2.73)	0.283
> 40	55 (100.00)	0 (0.00)	1.00		1.00	
Sex						
Male	169 (96.02)	7 (3.98)	0.92 (0.33–2.51)	0.866		
Female	237 (96.34)	9 (3.66)	1.00		1.00	
Level of education						
Diploma	18 (90.00)	2 (10.00)	0 (0–Infinity)	0.998		
Bachelor's degree	276 (95.5)	13 (4.50)	0 (0–Infinity)	0.998		
Master's degree	99 (99.00)	1 (1.00)	0 (0–Infinity)	0.998		
PhD	13 (100.00)	0 (0.00)	1.00		1.00	
Professional field						
Specialist	79 (100.00)	0 (0.00)	905284692.50 (0–Infinity)	0.998		
General practitioner	77 (100.00)	0 (0.00)	905285034.07 (0–Infinity)	0.998		
Nursing	169 (93.37)	12 (6.63)	0.6 (0.13–2.77)	0.512		
Midwifery	34 (94.44)	2 (5.56)	0.72 (0.1–5.39)	0.752		
Laboratory technician	47 (95.92)	2 (4.08)	1.00		1.00	
Years of experience						
0–5	270 (95.41)	13 (4.59)	0 (0–Infinity)	0.998		
6–10	112 (97.39)	3 (2.61)	0 (0–Infinity)	0.998		
> 10	24 (100.00)	0 (0.00)	1.00			
Monthly income (USD)*						
200–500	281 (96.23)	11 (3.77)	2.79 (1.37–5.7)	0.005*	3.23 (0.69–15.07)	0.136
501–1000	53 (92.98)	4 (7.02)	2.76 (1.1–6.94)	0.031*	0.49 (0.09–2.72)	0.413
> 1000	72 (98.63)	1 (1.37)	1.00		1.00	

*Note:* Some categories with very small cell counts were merged during multivariable analysis to improve estimate stability and avoid separation‐related inflation of odds ratios. * Indicates variable.

**Table 7:** Factors associated with preventive practices regarding needlestick injuries among healthcare workers in Mogadishu, Somalia.

	Practice level					
Variables	**Good, *n* (%)**	**Poor, *n* (%)**	OR (95% CI)	*p* value	AOR (95% CI)	*p* value
Age (years)*						
18–30	108 (39.56)	165 (60.44)	0.35 (0.19–0.63)	0.001*	0.3 (0.15–0.62)	0.001
31–40	45 (47.87)	49 (52.13)	0.48 (0.24–0.96)	0.039*	0.45 (0.22–0.94)	0.033
> 40	36 (65.45)	19 (34.55)	1.00		1.00	
Sex*						
Male	69 (39.20)	107 (60.80)	0.68 (0.46–1)	0.052	0.65 (0.41–1.01)	0.055
Female	120 (48.78)	126 (51.22)	1.00		1.00	
Level of education						
Diploma	8 (40.00)	12 (60)	1.07 (0.25–4.460)	0.930		
Bachelor's degree	128 (44.29)	161 (55.71)	1.27 (0.41–3.98)	0.679		
Master's degree	48 (48.00)	52 (52.00)	1.48 (0.45–4.83)	0.519		
PhD	5 (38.46)	8 (61.54)	1.00		1.00	
Professional field*						
Specialist	41 (51.9)	38 (48.1)	3.33 (1.51–7.31)	0.003*	2.67 (1.01–7.05)	0.048
General practitioner	33 (42.86)	44 (57.14)	2.31 (1.05–5.11)	0.038*	2.2 (0.96–5.06)	0.064
Nursing	86 (47.51)	95 (52.49)	2.79 (1.37–5.70)	0.005*	3.29 (1.58–6.86)	0.002
Midwifery	17 (47.22)	19 (52.78)	2.76 (1.1–6.94)	0.031*	3.39 (1.27–9.04)	0.015
Laboratory technician	12 (24.49)	37 (75.51)	1.00		1.00	
Years of experience						
0–5	118 (41.7)	165 (58.3)	1 (0.43–2.33)	0.998		
6–10	61 (53.04)	54 (46.96)	1.58 (0.65–3.85)	0.313		
> 10	10 (41.67)	14 (58.33)	1.00			
Monthly income (USD)*						
200–500	125 (42.81)	167 (57.19)	0.73 (0.44–1.22)	0.227	1.34 (0.61–2.94)	0.466
501–1000	27 (47.37)	30 (52.63)	0.88 (0.44–1.75)	0.707	1.20 (0.51–2.87)	0.675
> 1000	37 (50.68)	36 (49.32)	1.00		1.00	

*Note:* * Indicates variable.

## 5 Discussion

This study provides important insight into the knowledge, attitudes, and practices related to needlestick injuries (NSIs) among healthcare workers in Mogadishu, Somalia. The findings reveal a clear gap between relatively high levels of knowledge and generally positive attitudes, on the one hand, and weaker adherence to preventive practices on the other. This pattern suggests that awareness of occupational risks does not necessarily translate into consistent safe behavior in everyday clinical practice.

Overall, most participants demonstrated good knowledge of NSIs, particularly regarding the transmission of blood‐borne infections such as hepatitis B virus (HBV), hepatitis C virus (HCV), and human immunodeficiency virus (HIV). This finding is consistent with reports from other low‐ and middle‐income countries, where healthcare workers generally show good awareness of the infectious risks associated with sharps injuries [18, 19, 20]. However, important knowledge gaps remained, especially in relation to post‐exposure prophylaxis (PEP) and the appropriate timing of follow‐up testing after exposure. Similar deficiencies have been reported in studies from Sudan, Nepal, and India, where healthcare workers were aware of NSI risks but had limited knowledge of appropriate post‐exposure management [20, 21, 22]. These findings suggest that general awareness alone is insufficient and that targeted training should place greater emphasis on practical post‐exposure procedures.

Attitudes toward NSI prevention were largely positive. Most healthcare workers expressed concern about occupational exposure and acknowledged that needlestick injuries are preventable. This is broadly consistent with studies from Ethiopia, Pakistan, and Saudi Arabia, which also reported favorable attitudes toward infection prevention among healthcare workers [13, 21, 23]. However, a substantial proportion of participants still believed that patient care should take priority over healthcare worker safety. This may reflect a workplace culture in which patient outcomes are emphasized more strongly than occupational protection, a pattern that has also been described in other healthcare settings [23, 24]. Such attitudes could contribute to inconsistent adherence to safety procedures, even when healthcare workers are aware of the risks.

Attitudes toward needlestick injury prevention were generally positive across participant groups. However, the regression analysis for attitude was affected by the small number of participants reporting negative attitudes. This resulted in sparse and, in some categories, zero cell counts, producing unstable and imprecise estimates. Therefore, the observed associations should be interpreted cautiously and should not be considered definitive evidence of independent relationships. Although some differences were observed in the unadjusted analysis, these were not consistently retained after adjustment. Future studies involving larger samples and more balanced outcome groups are needed to provide more reliable estimates of factors associated with healthcare workers’ attitudes [14, 15].

Despite favorable knowledge and attitudes, preventive practices related to NSIs were suboptimal. Fewer than half of the participants demonstrated good preventive practices, and underreporting of injuries, together with delayed or inadequate uptake of PEP, emerged as major concerns. Underreporting of NSIs has been widely documented in previous studies and has been linked to factors such as perceiving the exposure as insignificant, fear of blame, lack of clear reporting systems, and limited awareness of institutional procedures [20, 22, 25]. In the present study, many participants considered some exposures insignificant, which may indicate a gap between understanding the general risk of NSIs and recognizing the importance of immediate and appropriate action after exposure. These findings highlight the need to strengthen reporting systems, promote a non‐punitive safety culture, and improve access to occupational health services.

Age‐related differences in preventive practice were also observed. Younger healthcare workers aged 18–30 years were significantly less likely to follow recommended safety practices than older colleagues. This finding is in line with earlier studies reporting lower compliance with safety measures among less experienced healthcare workers [19, 25]. However, the reasons for this pattern were not directly assessed in the present study. Possible explanations may include differences in clinical experience, practical training, confidence in handling occupational exposure, or perception of risk. Regardless of the underlying cause, this finding suggests that younger and early‐career healthcare workers may benefit from closer supervision, structured mentorship, and continuous practical training on infection prevention and post‐exposure management [19, 25, 26].

Professional differences in preventive practice were another important finding. Nurses and specialists showed better adherence to preventive practices than laboratory technicians. This pattern may reflect differences in job roles, clinical responsibilities, exposure settings, access to training, or institutional reinforcement of infection‐prevention measures across professional groups. Because these factors were not directly measured in the current study, these explanations should be interpreted as possible rather than definitive. Nevertheless, the finding suggests that preventive strategies should not be applied uniformly across all professions. Instead, role‐specific training and more consistent occupational safety support may be needed to address the different exposure contexts faced by various healthcare worker groups [19, 27, 28, 29].

The gap observed between knowledge, attitudes, and practices in this study is consistent with evidence from several other settings showing that awareness of needlestick injury prevention does not automatically lead to safe behavior [22, 23, 24, 27]. This pattern underscores the importance of moving beyond knowledge‐based interventions alone. Effective prevention is likely to require a broader approach that combines regular training, clear reporting mechanisms, supportive supervision, availability of protective equipment, timely access to PEP, and strong institutional commitment to occupational safety. In resource‐limited settings such as Somalia, strengthening workplace safety culture and ensuring practical support for healthcare workers may be especially important for reducing the burden of NSIs.

## 6 Limitations of the Study

This study has several limitations that should be considered when interpreting the findings. First, the cross‐sectional design does not allow causal relationships to be established between participant characteristics and their knowledge, attitudes, or preventive practices regarding needlestick injuries. The identified relationships should therefore be interpreted as associations rather than causal effects.

Second, the findings were based on self‐reported responses rather than direct observation of workplace practices or verification through occupational exposure records. The results may therefore have been influenced by recall and social desirability bias. Participants may have overreported desirable preventive practices or underreported unsafe behaviors, needlestick injuries, and delays in seeking post‐exposure care. Consequently, the reported practices may not fully reflect actual behavior in clinical settings.

Third, some regression analyses, particularly the model examining attitudes, were affected by sparse data because only a small number of participants reported negative attitudes. Several categories contained very small or zero cell counts, resulting in unstable and imprecise estimates. Although conceptually similar categories were combined where appropriate, the adjusted estimates should still be interpreted cautiously and should not be considered definitive evidence of independent associations.

Fourth, although healthcare workers from both public and private facilities were included, the study was conducted only in Mogadishu. The findings may therefore not be fully generalizable to healthcare workers in rural areas or other regions of Somalia, where healthcare infrastructure, occupational safety services, and resource availability may differ.

Despite these limitations, the study provides valuable context‐specific evidence regarding knowledge, attitudes, and preventive practices related to needlestick injuries among healthcare workers in Mogadishu. It also highlights important gaps in reporting practices and post‐exposure management that require greater institutional attention.

## 7 Conclusion

Healthcare workers in Mogadishu demonstrated generally good knowledge and positive attitudes toward needlestick injury prevention, but preventive practices remained inadequate. Fewer than half of the participants reported good preventive practices, while underreporting of injuries and delayed or inadequate uptake of post‐exposure prophylaxis remained important concerns. Preventive practices differed across age and professional groups, with younger healthcare workers reporting poorer practices. These findings highlight the need to strengthen injury‐reporting systems, improve access to post‐exposure services, and provide targeted occupational safety interventions. Associations derived from models affected by sparse data, particularly the attitude model, should be interpreted cautiously.

## Author Contributions

A.H.E. initially conceived the study. The study design was collaboratively developed by the A.H.E., M.M.A., M.M.H., and N.I.D. All four authors contributed to material preparation, data collection, and analysis. A.H.E. and M.M.A. were primarily responsible for data analysis and interpretation. A.H.E. drafted the initial version of the manuscript, and all the authors participated in revising, reviewing, and refining the subsequent versions.

## Ethics Statement

This study was conducted in compliance with ethical standards to ensure the rights and well‐being of all the participants. Approval for the study was granted by the Institutional Review Board (IRB) at SIMAD University in Mogadishu, Somalia, as indicated by the approval letter dated July 01, 2024 (reference number: 2024/SU‐IRB/FMHS/P0060). In line with this approval, all the participants provided informed consent prior to their involvement. They were thoroughly informed about the study's purpose, their right to confidentiality, and their freedom to withdraw from the study at any time, without any negative consequences.

## Consent

The authors have nothing to report.

## Conflicts of Interest

The authors declare no conflicts of interest.

## Transparency Statement

All authors have read and approved the final version of the manuscript. Abdullahi Hassan Elmi had full access to all of the data in this study and takes complete responsibility for the integrity of the data and the accuracy of the data analysis.

## Data Availability

The data that support the findings of this study are available from the corresponding author upon reasonable request.
